# Subjective cognitive decline in patients with migraine and its relationship with depression, anxiety, and sleep quality

**DOI:** 10.1186/s10194-017-0779-1

**Published:** 2017-07-25

**Authors:** Sun Hwa Lee, Yeonwook Kang, Soo-Jin Cho

**Affiliations:** 1 Department of Neurology, Hallym University Dongtan Sacred Heart Hospital, 7, Keunjaebong-gil, Hwaseong-si, Gyeonggi-do 445-907 South Korea; 2 Department of Psychology, Hallym University, Chuncheon, Korea

**Keywords:** Subjective cognitive decline, Migraine, Anxiety, Depression, Sleep

## Abstract

**Background:**

Cognitive decline is a major concern in patients with migraine. Depression, anxiety, and/or poor sleep quality are well-known comorbidities of migraine, but available evidence on the subjective cognitive decline (SCD) is limited. This study aimed to investigate the presence and frequency of SCD and its relationship with anxiety, depression and sleep quality in patients with migraine.

**Methods:**

We enrolled patients with migraine who scored within the normal range of the Korean-Mini Mental State Examination and the Korean-Montreal Cognitive Assessment. Using the Subjective Cognitive Decline Questionnaire (SCD-Q), participants with ≥7 were assigned to the SCD group. The Headache Impact Test-6, Generalized Anxiety Disorder-7, Patient Health Questionnaire-9, and Pittsburgh Sleep Quality Index were used and analyzed between the two groups.

**Results:**

A total of 188 patients with migraine, aged 38.1 ± 9.9 years, were enrolled. The mean SCD-Q score was 6.5 ± 5.5, and 44.7% of participants were identified as SCD. Migraineurs with SCD reported higher headache pain intensity and headache impact, as well as greater prevalence of anxiety, depression, reduced quality of sleep, and shorter sleep duration during weekdays compared to migraineurs without SCD. There were no significant differences in terms of age, sex, migraine type (chronic/episodic), medication, or sleep duration during weekends between the two groups. Upon multivariate logistic analysis adjusted for age, sex, headache characteristics, and psychological variables, depression was associated with increased risk of SCD (Odds ratio 1.31, 95% confidence interval 1.16–1.49) and sleep duration during weekdays was associated with decreased risk of SCD (Odds ratio 0.66, 95% confidence interval 0.44–0.97).

**Conclusions:**

A non-negligible number of patients with migraine complained of SCD. Depression and short sleep duration during weekdays were related to SCD among adult migraineurs.

## Background

Migraine is a common and disabling neurological disorder which leads to reduced quality of life and significant functional impairments [1]. In South Korea, a study investigating the prevalence of migraine over the course of 1 year reported that approximately 9.2% of women and 2.9% of men suffer from the disease [2]. The burden of migraine is compounded by other co-occurring disorders, including psychiatric disorders, sleep problems, epilepsy, and stroke [3]. Thus, it is important to identify manageable comorbidities for effective treatment of migraine.

There is contradictory evidence of the association between migraine and cognitive impairment. Some case-control studies reported that patients with migraine exhibit poor psychomotor speed [4], attention [5], and verbal memory [6] performance compared to non-migraineurs, even during postictal periods. Conversely, several longitudinal and cross-sectional studies have shown that migraine is not associated with cognitive dysfunction or decline and may even associate with better function or less decline [7–9]. Despite such inconsistent research findings on objective cognitive impairment, it is common that patients with migraine complain cognitive impairment in clinical practice. The perceptions of cognitive abilities are driven by comorbid symptoms rather than actual cognitive decline in patients with chronic pain. Therefore, cognitive problems in migraineurs is worthy to be approached from the assessment of complaints and its association with other comorbidities [10, 11].

Subjective cognitive decline (SCD), also known as “subjective memory impairment” in early studies, refers to any self-perceived or subjectively experienced worsening of cognitive function in the absence of impaired performance on cognitive tests [12]. SCD may represent the earliest symptoms of cognitive decline or preclinical Alzheimer’s disease (AD) and is also associated with psychological factors such as depression and anxiety in cognitively healthy elders [13] and cerebrovascular disease [14–19]. Multiple factors seem to contribute to the development of SCD; however, most studies on SCD are on the elderly population. Some investigations on healthy adults report that memory complaints are common, regardless of age, and seem to be related to negative affect [20, 21]. However, these studies focused only on “memory complaints” while difficulties in other cognitive domains, such as attention or executive function are also common in patients with migraine.

There is strong evidence indicating an association between migraine and several psychiatric conditions, such as mood and anxiety disorders [22, 23]. In a study using cluster analysis for patients with chronic migraine, patients with high levels of affective temperamental dysregulation showed a higher risk of suicidal behavior [24]. Recent studies also supported genome wide association between migraine and bipolar disorder, therefore, it is important to identify manageable psychiatric comorbidities for effective treatment and improving life quality among migraineurs [25, 26]. However, an association between SCD and comorbid symptoms or pain severity has not yet been investigated with relevant cognitive tests among patients with migraine.

We hypothesized that the migraineurs with SCD would complain of more severe pain and exhibit greater psychiatric comorbidities than individuals with migraine without SCD. Thus, the aim of the present study was to investigate the presence and frequency of SCD in patients with migraine, as well as to analyze the association of SCD with clinical features and headache impact, anxiety, depression and quality of sleep.

## Methods

### Participants

**Fig. 1:**
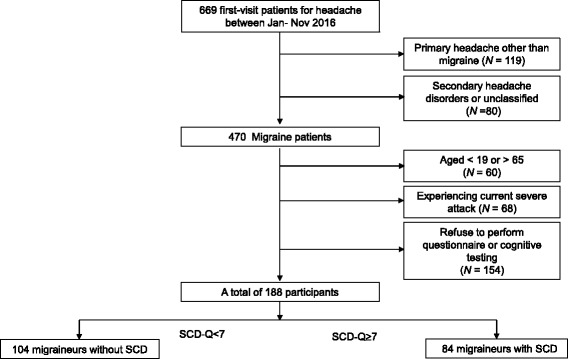
Flow diagram of sample selection

The present study was based on a retrospective review of headache registry records. Records were of first visits for headache between January and November 2016 at the Department of Neurology Headache Clinic at the Hallym University Dongtan Sacred Heart Hospital. Inclusion criteria were as follows: 1) diagnosis of episodic migraine with or without aura, or diagnosis of chronic migraine, 2) completion of cognitive testing and questionnaires, and 3) normal performance on cognitive tests based on normative data, stratified by gender, age, and education level. Exclusion criteria were as follows: 1) diagnosis of primary headache disorder other than migraine, 2) secondary headache disorders other than medication-overuse headache or unclassified headache, 3) aged under 18 years or older than 65 years, 4) patients with migraine who were experiencing a severe attack, 5) refusal to perform cognitive testing or questionnaires (Fig. 1). The diagnosis of headache disorders was based on the beta version of the International Classification of Headache Disorder, 3rd edition [27]. Migraine with and without aura was considered as episodic migraine.

The participants completed questionnaires about SCD, psychological status, and headache impact. They also underwent cognitive screening tests for the evaluation of comorbid disorders. All participants were fully informed of the purpose and procedures of the study and were assured confidentiality.

The study protocol was reviewed and approved by the Institutional Review Board of the Hallym University Dongtan Sacred Heart Hospital. The Institutional Review Board allowed the process of informed consent to be waived due to the post-hoc analysis of collected clinical data.

### Assessments and procedures

Demographic data such as age, sex, headache type (chronic/episodic and with aura/without aura/chronic/probable), frequency of attack (per month), maximum pain duration (in hours), and number of tablets (per month) were collected and assessed when the participants first visited the clinic. Participants scored their subjective perception of average pain intensity on a Visual Analogue Scale (VAS) [28]. Headache impact was scored using the Headache Impact Test-6 (HIT-6), [29] which included six questions to measure headache-related burden. The questions evaluated pain, social functioning, role functioning, vitality, cognitive functioning, and psychological distress. Each item was answered on a five-point scale (6 = never, 8 = rarely, 10 = sometimes, 11 = very often, 13 = always). Total scores ranged from 36 to 78, and higher scores indicated greater impact.

Two cognitive screening tests, the Korean-Mini Mental State Examination (K-MMSE) [30] and the Korean-Montreal Cognitive Assessment (K-MoCA) [31], were administered by a clinical neuropsychologist. The K-MMSE included items assessing orientation (5 points for time- and 5 points for place-orientation), memory (3 points for immediate- and 3 points for delayed-recall), serial subtractions (5 points), language ability (2 points for naming, 3 points for oral command comprehension, and 1 point each for repetition, reading, and writing), and visuospatial ability (1 point). The K-MoCA consisted of the following sequential items: alternate trail making (1 point), copying a cube (1 point) and drawing a clock (3 points) (to assess visuo-construction skills), naming (3 points), attention (6 points), sentence repetition (2 points), verbal fluency (1 point), abstraction (2 points), delayed recall (5 points), and orientation (6 points). An interval of 10 min was given between the two screening tests. Participants were administered the K-MMSE and K-MoCA in counterbalanced fashion and the order of administration did not affect test performance. The total scores for both tests ranged from 0 to 30, and higher scores indicated greater cognitive function.

The SCD questionnaire (SCD-Q) [32] was used to assign participants to either the “no SCD group” or the “SCD group”. The SCD-Q consisted of 24 items assessing three cognitive areas, including memory (11 items), language (6 items), and executive (7 items) domains. The response to each question was restricted to “yes/no” based on one’s perceived decline in each item. The total SCD-Q score ranged from 0 to 24, with higher scores indicating greater subjective perception of one’s cognitive decline over the past 2 years. The cutoff value was ≥7.

The following self-report questionnaires were also administered to reveal clinical characteristics often complained by patients with migraine.

*The Generalized Anxiety Disorder-7 (GAD-7)*, [33] which included seven items pertaining to the Diagnostic and Statistical Manual of Mental Disorders, 5th Edition (DSM-IV) criteria for a generalized anxiety disorder [34]. Each item was rated on a four-point scale, from 0 to 3 (0 = never, 1 = several days, 2 = more than half the time, and 3 = nearly every day). Items were rated based on occurrence over the previous 2 weeks. The total scores ranged from 0 to 21 and the cutoff score was 5 [35].

*The Patient Health Questionnaire-9 (PHQ-9)* [36], which included nine items pertaining to the DSM-IV criteria for a Major Depressive Disorder (MDD) [34]. Each item was rated on a four-point scale, from 0 to 3 (0 = never, 1 = several days, 2 = more than half the time, and 3 = nearly every day). Items were rated based on occurrence over the previous 2 weeks. The total scores ranged from 0 to 27. A cutoff score of 7 was considered sensitive and specific to detect MDD in a patient population with migraine [37].

*The Pittsburgh Sleep Quality Index (PSQI)* [38] was used to measure the quality and patterns of sleep over the preceding 4 weeks. The PSQI differentiated “poor” from “good” sleep by measuring the following seven areas: subjective sleep quality, sleep latency, sleep duration, habitual sleep efficiency, sleep disturbances, use of sleeping medication, and daytime dysfunction. Scoring was based on a 0–3 Likert scale and the sum of scores for these seven components yielded one global score ranged from 0 to 21. A total score of >5 indicated poor subjective sleep quality. In addition to the PSQI, participants were also asked to record the average amount of sleep they achieved during weekdays and weekends.

### Data analysis

Microsoft Excel 2010 and PASW statistics for Windows (version 18.0; SPSS Inc., Chicago, IL) were used for data analysis. Test results are reported as mean and standard deviations (SD) for normally distributed continuous variables. An *independent-sample t-test* with *Levene’s test* for equal of variances was conducted for parametric data. *Pearson’s chi-square test* was performed for categorical variables. Correlational analyses were performed using *Spearman’s rank correlation* coefficient (*r*_*s*_). Multivariate logistic regression analysis was also conducted to investigate whether SCD may be predicted by anxiety, depression and sleep quality in patient with migraine.

## Results

Among a total of 669 first-visit patients for headache between January to November 2016, 481 patients were excluded due to following criteria (Fig. 1): having primary or secondary headache disorders other than migraine (*N* = 199), age criteria (*N* = 60), experiencing severe pain (*N* = 68), and refuse to perform questionnaire or cognitive testing (*N* = 154). Among migraineurs, none was excluded due to abnormal range of performance on both screening tests. Amongst the total of 470 migraine patients, the age of included patients (*N* = 188, mean age = 38.09 ± 9.92) were significantly younger than the excluded patients (*N* = 282, mean age = 40.68 ± 14.43, *p* = 0.032), because those who were aged <19 or >65 were excluded. However, the percentage of gender did not differ between excluded versus included migraine patients (*p* = 0.610).

A total of 188 participants with migraine, aged 38.1 ± 9.9 years, were included. The mean SCD-Q score was 6.5 (SD = 5.5), with 84 patients (44.7%) scoring higher than 7. Thus, 44.7% of participants were diagnosed as SCD in this study. Among the 188 participants, 106 (56.4%) scored higher than the cutoff on the GAD-7, 98 (52.1%) scored higher than the cutoff on the PHQ-9, and 154 (81.9%) scored higher than the cutoff on the PSQI.

Demographic and headache-related clinical data are shown in Table 1. An *independent-sample t-test* and *Pearson’s Chi-square* test did not reveal differences between groups in terms of age, sex, migraine type, pain duration, and medication. However, significant differences were found for pain intensity on the VAS and headache impact on the HIT-6. There were no significant differences between groups on both cognitive screening tests─the K-MMSE and K-MoCA (Table 1). Moreover, no group differences were found in any sub-items of both tests.

**Table 1:** Demographical and clinical characteristics of no-SCD versus SCD groups

	No-SCD (*n* = 104)	SCD (*n* = 84)	*p-value*
Age	37.3 ± 9.8	39.1 ± 10.0	0.228
Sex (male/female)	24/80	24/60	0.390
Episodic/chronic headache	84/20	65/19	0.569
Migraine types (with aura/without aura/chronic/probable)	5/75/20/4	8/58/18/0	0.179
Frequency of attacks per month	8.9 ± 8.1	10.2 ± 7.2	0.236
Maximum pain duration in hours	35.3 ± 54.0	31.3 ± 31.3	0.542
Number of tablets per month	4.3 ± 5.9	5.6 ± 6.7	0.169
Pain intensity on VAS	6.6 ± 1.8	7.2 ± 1.5	0.024
HIT-6	59.2 ± 7.6	62.3 ± 7.0	0.005
K-MMSE	29.0 ± 1.5	28.9 ± 1.6	0.566
K-MoCA	27.5 ± 1.9	27.5 ± 2.0	0.966

*VAS* Visual Analogue Scale, *HIT-6* Headache Impact Test-6, *SCD* subjective cognitive decline, *K-MMSE* Korean-Mini Mental State Examination, *K-MoCA* Korean-Montreal Cognitive Assessment

Of the 84 migraineurs with SCD, 56 (66.7%) scored higher than the cutoff on the GAD-7, 59 (70.2%) scored higher than the cutoff on the PHQ-9, and 73 (86.9%) scored higher than the cutoff on the PSQI. Group differences between SCD and no-SCD groups in terms of anxiety, depression, and sleep quality are shown in Table 2. Significant differences were found in anxiety (GAD-7), depression (PHQ-9), sleep quality (PSQI), and average sleep (in hours) during weekdays. However, there was no significant group difference in terms of the average amount of sleep achieved during weekends.

**Table 2:** Anxiety, depression, sleep quality, and average sleeping hours during weekdays and weekends of no-SCD versus SCD groups

	No-SCD (*n* = 104)	SCD (*n* = 84)	*p-value*
GAD-7	5.0 ± 3.9	8.2 ± 5.5	< 0.001
PHQ-9	5.7 ± 4.0	10.4 ± 5.6	< 0.001
PSQI	8.5 ± 3.8	10.6 ± 4.3	< 0.001
Average Sleeping hours during weekdays	6.5 ± 1.1	6.0 ± 1.3	0.008
Average Sleeping hours during weekends	7.6 ± 1.5	7.3 ± 2.0	0.335

*GAD-7* The Generalized Anxiety Disorder-7, *PHQ-9* The Patient Health Questionnaire-9, *PSQI* The Pittsburgh Sleep Quality Index, *SCD* subjective cognitive decline

SCD-Q scores were correlated with the depression on the PHQ-9 (*r*_*s*_ = 0.49, *p* < 0.001), anxiety on the GAD-7 (*r*_*s*_ = 0.34, *p* < 0.001), sleep quality on the PSQI (*r*_*s*_ = 0.33, *p* < 0.001), sleep duration during weekdays (*r*_*s*_ = −0.20, *p* = 0.007), VAS (*r*_*s*_ = 0.17, *p* = 0.017), and the frequency of headache attacks per month (*r*_*s*_ = 0.15, *p* = 0.047). However, SCD-Q scores were not significantly correlated with the HIT-6, age, maximum pain duration, medication, sleep duration during weekends, nor cognitive screening test scores (data not shown).

Upon multivariate logistic analysis adjusted for age, sex, headache characteristics, and psychological variables, depression was associated with increased risk of SCD (Odds ratio 1.31, 95% confidence interval 1.16–1.49) and sleep duration during weekdays was associated with decreased risk of SCD (Odds ratio 0.66, 95% confidence interval 0.44–0.97) (Table 3). As the level of depression on PHQ-9 increases by 1 unit, the likelihood of migraineurs with SCD increases by 0.272 times (b = 0.27, Wald χ^2^(1) = 17.50, *p* < 0.001). As the sleep duration during weekdays increases by 1 unit, the likelihood of migraineurs with SCD lower by 0.423 times (b = −0.423, Wald χ^2^(1) = 4.39, *p* = 0.036).

**Table 3:** Association of SCD with demographical and clinical variables: Multivariate logistic regression analyses

	OR (95% CI)	*p-value*
Age	1.03 (0.99–1.08)	0.131
Sex (male/female)	0.88 (0.40–1.95)	0.749
Episodic/chronic headache	0.97 (0.17–5.43)	0.968
Migraine types (with aura/without aura/chronic/probable)	0.73 (0.35–1.51)	0.392
Frequency of attacks per month	1.02 (0.95–1.09)	0.568
Maximum pain duration in hours	0.994 (0.983–1.006)	0.331
Number of tablets per month	1.02 (0.95–1.09)	0.590
Pain intensity on VAS	1.14 (0.90–1.45)	0.268
HIT-6	1.00 (0.94–1.07)	0.887
K-MMSE	1.02 (0.75–1.37)	0.919
K-MoCA	1.09 (0.85–1.38)	0.507
GAD-7	0.96 (0.86–1.07)	0.492
PHQ-9	1.31 (1.16–1.49)	<0.001
PSQI	0.92 (0.82–1.03)	0.161
Average Sleeping hours during weekdays	0.66 (0.44–0.97)	0.036
Average Sleeping hours during weekends	1.02 (0.77–1.34)	0.913

*VAS* Visual Analogue Scale, *HIT-6* Headache Impact Test-6, *SCD* subjective cognitive decline, *K-MMSE* Korean-Mini Mental State Examination, *K-MoCA* Korean-Montreal Cognitive Assessment, *GAD-7* The Generalized Anxiety Disorder-7, *PHQ-9* The Patient Health Questionnaire-9, *PSQI* The Pittsburgh Sleep Quality Index

## Discussion

We investigated SCD amongst the patients with migraine using a standard questionnaire with relevant cognitive testing. The main findings of this study were 1) SCD is relatively common in adult patients with migraine, 2) migraineurs with SCD reported severe headache pain severity and headache impact, 3) migraineurs with SCD were more depressed and anxious, while experiencing lowered sleep quality and sleep duration during weekdays were shorter, 4) depression on the PHQ-9 and sleep duration during weekdays were associated with SCD after adjusting demographic variables, headache related variables and psychological variables.

Previous studies found that patients with migraine showed normal range of performance on both the MMSE [39] and MoCA [40]. Similarly, none were excluded due to abnormal performance on screening tests among migraineurs in this study. Considering the mean SCD-Q scores of 3.2 (SD = 3.7) in normal controls in the previous study, the mean SCD-Q score of 6.5 (SD = 5.5) in migraineurs was relatively high [32]. Not negligible number of young adult migraineurs seemed to complaint cognitive decline and, to our knowledge, this is the first report to identify SCD in adult patients with migraines. Moreover, the proportion of SCD (44.2%) is comparable to common comorbidities of migraine such as poor quality of sleep (81.6%), anxiety (55.8%) and depression (52.1%).

SCD in preclinical AD received particular interest on aging studies since longitudinal data support SCD as a risk factor for future cognitive decline as well as AD dementia [14, 17, 41]. A review study on SCD suggested that the proposed age of onset in studies of preclinical AD is 60 years or older despite the age at SCD onset was not defined as a core criterion [42]. Unlikely to SCD in elderly, there is no conclusive evidence for that SCD or perceived forgetfulness in young adult is a risk factor for future cognitive impairment or dementia [43]. In studies of healthy adults, memory complaints are frequent regardless of age; however, the type of complaint varies and may be related to negative affect throughout adulthood [20, 21]. This study also suggested that SCD among migraineurs require more attention to psychological or sleep problem rather than close follow-up of cognitive decline.

Subjective pain is a common symptom in patients suffering from depression and in turn, chronic pain may trigger a depressive state [44, 45]. Interestingly, migraineurs with SCD complained of more severe difficulties in subjectively reported measures, such as pain intensity and headache impact, compared to migraineurs without SCD, but this association was not persistent on logistic analysis. This implies such sensitivity to perceived pain or subjective difficulties may be related to psychological impairments and/or sleep problem among migraineurs with SCD.

In line with previous studies, our data also revealed high levels of anxiety and depression in patients with migraine [46]. Not surprisingly, depression was significantly associated with SCD among migraineurs in this study. It is possible that SCD among migraineurs may either independently or conjointly account for the high levels of depression and anxiety. Moreover, biased perceptions of cognitive function may relate more to emotional state than objective ability, causing individuals to misinterpret or exaggerate problems by overestimating minor cognitive disruptions. According to Beck’s cognitive theory of depression, depression is associated with a negative view of oneself, environment, and future [47]. Increasing the awareness patients with migraine have of possible misperceptions could help them understand their neurological and psychological state more objectively.

Similar to a previous study, more than half of the total patients with migraine that were evaluated in the current study complained of poor sleep quality [48]; this percentage was even higher (86.9%) in migraineurs with SCD. This relationship may be explained by the high correlation between SCD-Q and PSQI scores. Indeed, a previous study revealed an association between perception of poor sleep quality and worse cognitive performance in healthy elderly individuals [49]. In the current study, the average amount of sleep achieved during weekdays for migraineurs with SCD was less than 6 h. This value is less than the recommended duration of 7 to 8 h for adults [50]. It has been reported that migraineurs who routinely sleep 6 h per night exhibit more severe headache patterns and sleep complaints than migraineurs who slept longer [51]. Thus, short sleep duration during weekdays may account for the poor perception of cognitive function and sleep quality observed in the current study.

Several limitations to this study should be addressed. The main limitations of the study would be that it is based on retrospective review of headache registry records. Therefore, this study may be biased in its sampling procedure and we cannot rule out the possibility that individuals who perceived SCD were more inclined to agree to cognitive testing. However, variables in this study were collected prospectively according to the registry and the difference between excluded and included patients was not significant in demographical characteristics such as gender. In addition, we used brief screening tests (i.e., K-MMSE and K-MoCA) as cognitive measures. These were employed as it is difficult to recruit a substantial number of patients willing to undergo comprehensive neuropsychological tests in clinical settings. Although there were no group differences in any sub-items of both tests, more detailed cognitive tests may help reveal subtle cognitive characteristics that might not yet exceed age-, sex-, and education-adjusted normal ranges in migraineurs with SCD. Lastly, future studies should include a healthy control group to provide better standards for understanding migraineurs with SCD.

## Conclusions

In conclusion, SCD seems relatively common in adult migraineurs in terms of both gender and migraine subtype. Migraineurs with SCD reported severe pain and impact of headache, were more depressed, anxious, perceived poorer sleep quality, and shorter sleep duration during weekdays than those without SCD. Depression and shorter sleep duration significantly associated to the presence of SCD in migraineurs after adjusting other variables.

## Abbreviations

DSM-IV: Diagnostic and Statistical Manual of Mental Disorders 5th Edition
GAD-7: Generalized Anxiety Disorder-7
HIT-6: Headache Impact Test-6
K-MMSE: Korean-Mini Mental State Examination
K-MoCA: Korean-Montreal Cognitive Assessment
MDD: Major Depressive Disorder
PHQ-9: Patient Health Questionnaire-9
PSQI: Pittsburgh Sleep Quality Index
SCD: Subjective Cognitive Decline
SCD-Q: SCD questionnaire
VAS: Visual Analogue Scale
